# Optimal image quality and radiation doses with optimal tube voltages/currents for pediatric anthropomorphic phantom brains

**DOI:** 10.1371/journal.pone.0306857

**Published:** 2024-07-22

**Authors:** Li-Guo Chen, Hung-Wen Kao, Ping-An Wu, Ming-Huei Sheu, Li-Chuan Huang

**Affiliations:** 1 Department of Medical Imaging, Hualien Tzu Chi Hospital, Buddhist Tzu Chi Medical Foundation, Hualien, Taiwan; 2 Department of Radiology, School of Medicine, Tzu Chi University, Hualien, Taiwan; 3 Department of Medical Imaging and Radiological Sciences, Tzu Chi University of Science and Technology, Hualien, Taiwan; Polytechnic University of Marche, ITALY

## Abstract

**Objective:**

Using pediatric anthropomorphic phantoms (APs), we aimed to determine the scanning tube voltage/current combinations that could achieve optimal image quality and avoid excessive radiation exposure in pediatric patients.

**Materials and methods:**

A 64-slice scanner was used to scan a standard test phantom to determine the volume CT dose indices (CTDI_vol_), and three pediatric anthropomorphic phantoms (APs) with highly accurate anatomy and tissue-equivalent materials were studied. These specialized APs represented the average 1-year-old, 5-year-old, and 10-year-old children, respectively. The physical phantoms were constructed with brain tissue-equivalent materials having a density of ρ = 1.07 g/cm^3^, comprising 22 numbered 2.54-cm-thick sections for the 1-year-old, 26 sections for the 5-year-old, and 32 sections for the 10-year-old. They were scanned to acquire brain CT images and determine the standard deviations (SDs), effective doses (EDs), and contrast-to noise ratios (CNRs). The APs were scanned by 21 combinations of tube voltages/currents (80, 100, or 120 kVp/10, 40, 80, 120, 150, 200, or 250 mA) and rotation time/pitch settings of 1 s/0.984:1.

**Results:**

The optimal tube voltage/current combinations yielding optimal image quality were 80 kVp/80 mA for the 1-year-old AP; 80 kVp/120 mA for the 5-year-old AP; and 80 kVp/150 mA for the 10-year-old AP. Because these scanning tube voltages/currents yielded SDs, respectively, of 12.81, 13.09, and 12.26 HU, along with small EDs of 0.31, 0.34, and 0.31 mSv, these parameters and the induced values were expediently defined as optimal.

**Conclusions:**

The optimal tube voltages/currents that yielded optimal brain image quality, SDs, CNRs, and EDs herein are novel and essentially important. Clinical translation of these optimal values may allow CT diagnosis with low radiation doses to children’s heads.

## Introduction

Pediatric computerized tomography (CT) examination requires rapid scanning, which can reduce the need for tranquilizers and decrease artifacts caused by turbulence. Although higher radiation doses can improve image quality and the contrast-to-noise ratio (CNR) [1–3], the radiation sensitivity of children’s cells is higher than that of adults and the damage caused by radiation is accumulative [4–9]. Radiologists routinely use scanning tube voltage/current settings recommended by the vendor to ensure that the CT images are suitable for clinical diagnosis [10]; nevertheless, the suitability of these scanning parameters in terms of ensuring optimal radiation doses for children remains unknown [11]. Therefore, optimal tube voltages/currents that take into account both optimal images and low radiation doses are important in pediatric CT [12–14].

In this regard, optimal scanning tube voltages/currents are needed to avoid excessive radiation and the resultant deterministic or genetic effects in pediatric cells [15]. The International Commission on Radiological Protection recommends that diagnostic images be obtained at the optimal dose by minimizing the risk and making it reasonable [16–18]. The International Atomic Energy Agency found that the pediatric volume CT dose index (CTDI_vol_), a dose monitoring indicator, shows significant differences across various countries, indicating the need to optimize pediatric CT in many developing countries [19–22]. The optimal tube voltages/currents for children’s brain CT images in relation to CTDI_vol_, image-noise standard deviations (SDs), effective doses (EDs), and CNRs are rarely known because repeated radiations to a human being within a short time are prohibited. Thus, optimization of these parameters for pediatric brain CT imaging is particularly important, yet difficult.

Anthropomorphic phantoms (APs) serve as a useful tool for simulating human subjects in radiation dosimetry; they are particularly useful for procedures involving repeated scans within a short period of time to study radiation exposure in diagnostic imaging procedures [23, 24]. Thus, APs offer an alternative for the determination of tube voltages/currents that can yield optimal SDs and CNRs as well as reduce the impact of radiation on children’s brains. Although many investigations have used standard pediatric APs to analyze tube voltages/currents, SDs, and CNRs [25–28], these parameters are not sufficient to achieve the As Low As Reasonably Achievable (ALARA) principle [29]; in other words, they were not optimal.

Therefore, we hypothesized that APs could be used to allow repeated testing of tube voltages/currents for acquiring optimal CT images and related measurements. This approach would allow the determination of the optimal tube voltages/currents that can yield CT images suitable for diagnosis without excessive radiation exposure. To test this hypothesis, we aimed to use APs representing 1-, 5-, and 10-year-old brains and determine the optimal tube voltages/currents that could yield sufficient CT image quality while avoiding excessive radiation exposure. We expect that the clinical translation of these optimal tube voltage/current values will reduce the impact of diagnostic radiation on children’s brains.

## Materials and methods

### Experimental protocols for APs

**Fig 1:**
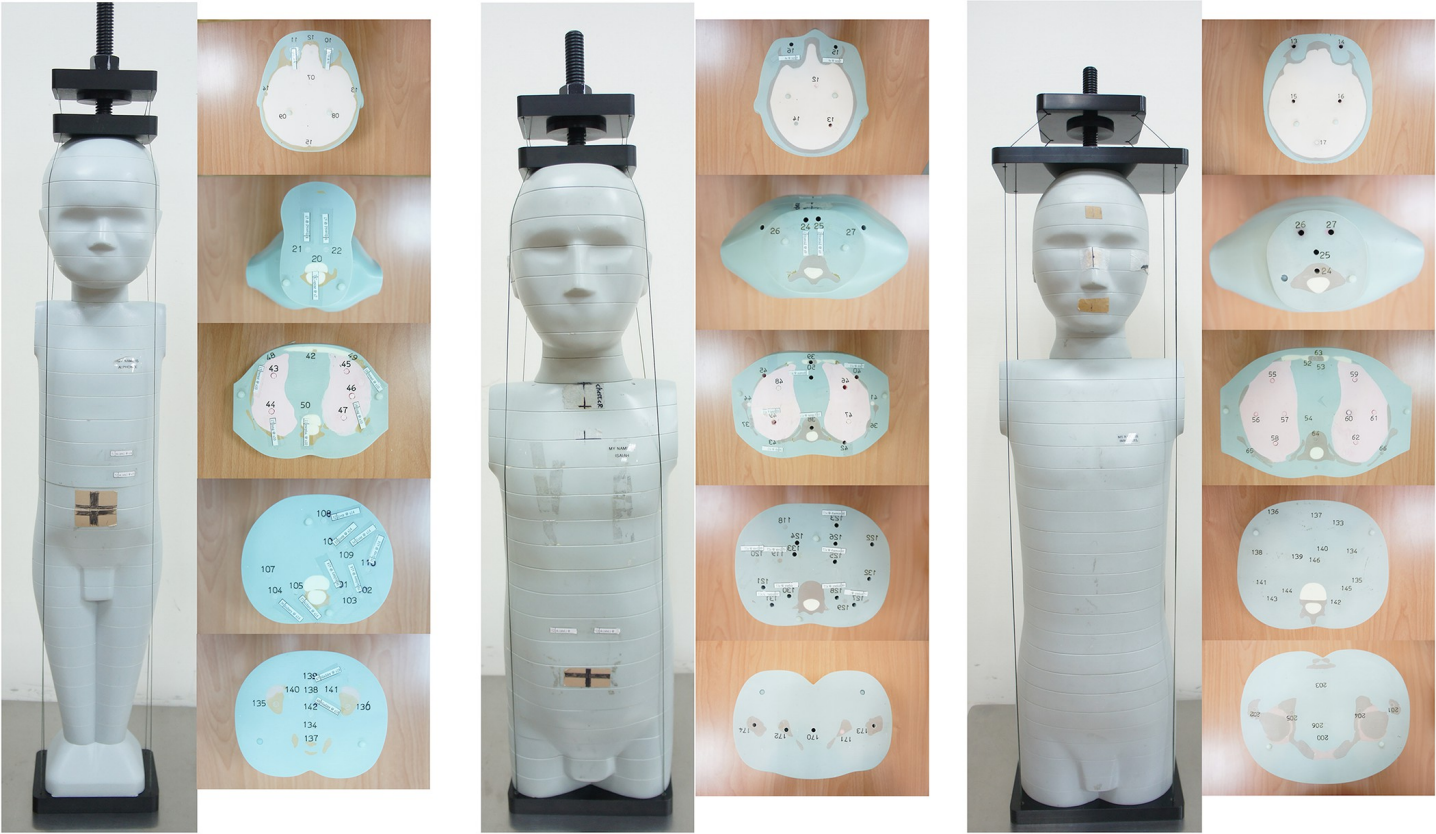
The three anthropomorphic phantoms (APs) serving as representatives of 1- (left), 5- (middle) and 10-year-old (right) children.

Three APs (ATOM; CIRS, Norfolk, VA), which are also called anthropomorphic prostheses, representative of 1-, 5-, and 10-year-old children were obtained (Fig 1). These physical phantoms consisted of 22, 26, and 32, 2.54-cm-thick transverse sections, respectively, and contained six different tissue types: soft tissue (density, ρ = 1.05 g/cm^3^), spinal cord (ρ = 1.07 g/cm^3^), spinal discs (ρ = 1.15 g/cm^3^), lung (ρ = 0.20 g/cm^3^), brain (ρ = 1.07 g/cm^3^), and the physical density of a 1-year-old (ρ = 1.45 g/cm^3^), 5-year-old (ρ = 1.52 g/cm^3^), and 10-year-old (ρ = 1.56 g/cm^3^). CT images of the three AP heads were acquired by a 64-section multidetector row CT scanner (LightSpeed VCT; GE Healthcare, Inc, Milwaukee, WI), using successive scanning tube voltages of 80, 100, and 120 kVp. Each tube voltage was accompanied with a tube current of 10, 40, 80, 120, 150, 200, or 250 mA. The scanner configuration was 64*0.625 mm (gantry rotation time, 1 s; beam pitch, 0.984:1). The limited volume scan covered the following anatomic areas: centrum semiovale, corona radiata at the lateral ventricles, middle cranial fossa/skull base, and posterior fossa/palate. This study was limited to CT examinations for general brain diseases such as brain edema, brain infection, brain injury, cerebral hemorrhage, and brain tumors.

The reconstruction slice thickness was kept constant at 2.5 mm for the 21 different combinations. CT images were reconstructed by using the standard reconstruction filter in 512*512 matrixes with a pixel size of 1.367 mm. Subsequently, measurements were made to evaluate volume computed tomography indices (CTDI_vol_), dose-length-products (DLPs), and effective doses (EDs), noise levels (SDs), and CNRs (Tables 1 and 2).

**Table 1:** The 1-, 5-, and 10-year-old APs were scanned by 21 combinations of tube voltages/currents to obtain CTDI_vol_, DLP, EDs, SDs, and CNRs.

Tube voltage	Tube current	CTDI_vol_	1-year-old				5-years-old				10-years-old			
			DLP	EDs	SDs	CNRs	DLP	EDs	SDs	CNRs	DLP	EDs	SDs	CNRs
(kVp)	(mA)	(mGy)	(mGy-cm)	(mSv)	(HU)		(mGy-cm)	(mSv)	(HU)		(mGy-cm)	(mSv)	(HU)	
80	10	0.53	7.70	0.04	40.08	0.77	8.09	0.03	61.92	0.27	8.62	0.02	73.47	0.06
	40	2.11	30.80	0.15	18.54	1.02	32.38	0.11	23.25	0.79	34.49	0.08	24.41	0.86
	80	4.22	61.59	0.31	12.81	1.20	64.76	0.23	16.78	0.97	68.98	0.17	15.72	1.07
	120	6.33	92.39	0.46	10.67	1.24	97.14	0.34	13.09	1.02	103.47	0.25	14.24	1.06
	150	7.91	115.49	0.58	9.37	1.30	121.42	0.42	12.71	1.03	129.33	0.31	12.26	1.11
	200	10.55	153.98	0.77	8.22	1.31	161.89	0.57	10.75	1.08	172.44	0.41	10.59	1.17
	250	13.19	192.48	0.96	7.65	1.31	202.37	0.71	9.50	1.08	215.56	0.52	9.49	1.16
100	10	1.05	15.40	0.08	26.43	1.42	16.19	0.06	31.92	1.11	17.24	0.05	33.93	1.10
	40	4.22	61.59	0.34	12.49	1.72	64.76	0.26	15.63	1.46	68.98	0.19	16.90	1.53
	80	8.44	123.19	0.68	8.80	1.74	129.52	0.52	11.79	1.50	137.96	0.37	11.04	1.65
	120	12.66	184.78	1.02	7.16	1.83	194.27	0.78	9.43	1.53	206.93	0.56	9.01	1.73
	150	15.82	230.97	1.27	6.93	1.84	242.84	0.97	8.02	1.61	258.67	0.70	7.35	1.76
	200	21.10	307.96	1.69	5.82	1.87	323.79	1.30	7.21	1.63	344.89	0.93	7.54	1.77
	250	26.37	384.95	2.12	5.45	1.87	404.74	1.62	6.05	1.64	431.11	1.16	6.33	1.79
120	10	1.70	24.84	0.15	19.04	1.69	26.11	0.12	27.13	1.37	27.81	0.09	27.51	1.43
	40	6.81	99.34	0.61	10.76	1.95	104.45	0.48	12.77	1.69	111.25	0.34	12.30	1.90
	80	13.61	198.69	1.21	7.49	2.01	208.90	0.96	9.59	1.71	222.51	0.69	9.06	1.98
	120	20.42	298.03	1.82	6.11	2.05	313.34	1.44	7.69	1.82	333.76	1.03	8.06	2.01
	150	25.52	372.54	2.27	5.63	2.07	391.68	1.80	6.86	1.85	417.20	1.29	6.09	2.09
	200	34.03	496.72	3.03	4.76	2.08	522.24	2.40	5.89	1.86	556.27	1.72	5.62	2.09
	250	42.54	620.89	3.79	4.36	2.08	652.80	3.00	5.52	1.86	695.34	2.16	5.26	2.09

AP = anthropomorphic phantoms; CTDI_vol_ = volume computed tomography dose index; DLP = dose length product; ED = effective dose

SD = standard deviation; CNR = contrast-to-noise ratio.

**Table 2:** Tube voltage/current-induced ranges of changes in SDs, CNRs and EDs for the three age groups.

Tube kVp/mA	Ages	SDs (HU)	EDs (mSv)	CNRs
80/10-250	1-year-old	40.08–7.65	0.04–0.96	0.77–1.31
	5-year-old	61.92–9.50	0.03–0.71	0.27–1.08
	10-year-old	73.47–9.49	0.02–0.52	0.06–1.16
100/10-250	1-year-old	26.43–5.45	0.08–2.12	1.42–1.87
	5-year-old	31.92–6.05	0.06–1.62	1.11–1.64
	10-year-old	33.93–6.33	0.05–1.16	1.10–1.79
120/10-250	1-year-old	19.04–4.36	0.15–3.79	1.69–2.08
	5-year-old	27.13–5.52	0.12–3.00	1.37–1.83
	10-year-old	27.51–5.26	0.09–2.16	1.43–2.09

SD = standard deviation; CNR = contrast-to-noise ratio; ED = effective doses.

### Region of interest, CT number, SD, and CNR

**Fig 2:**
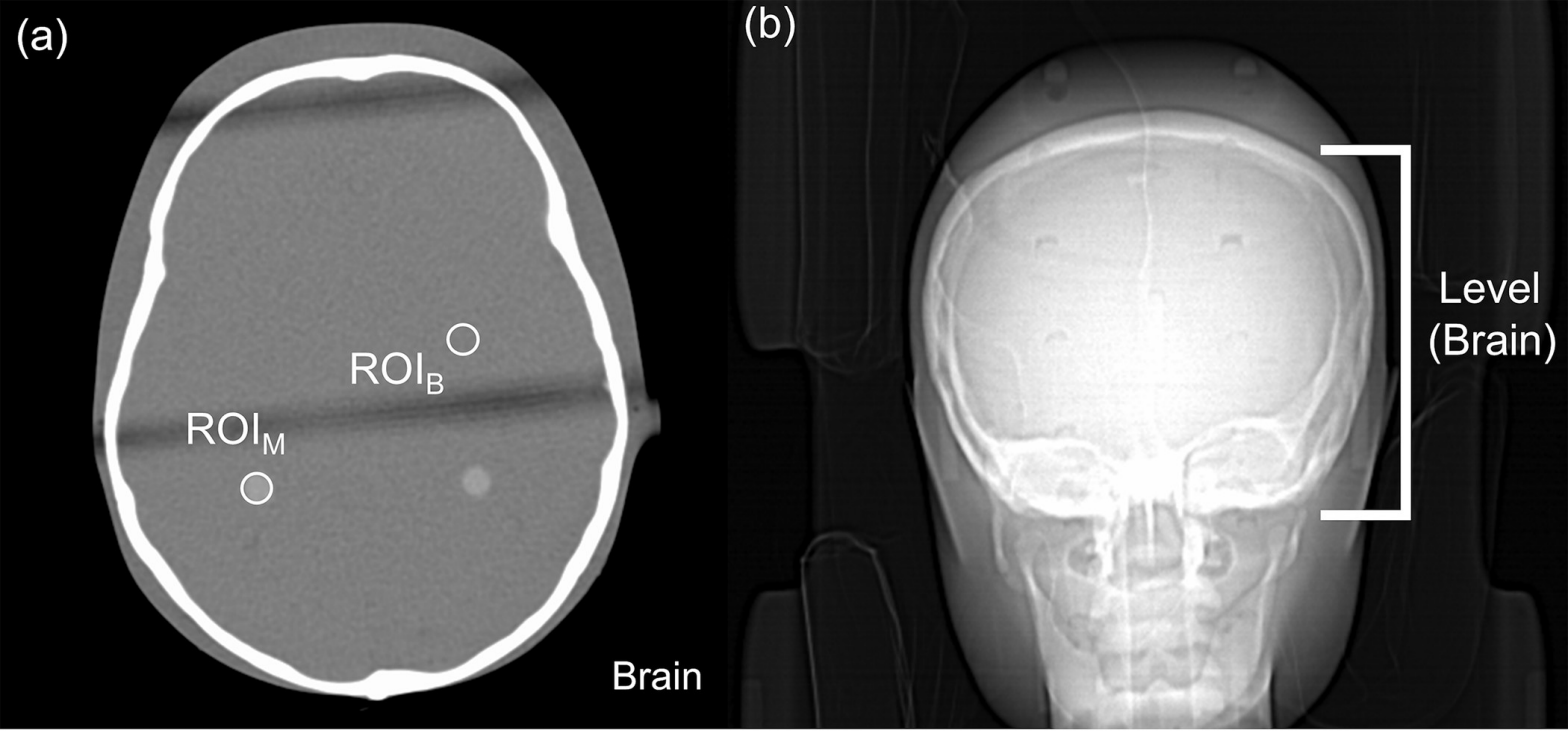
Determination of the axial image quality for the 1-year-old AP brain (window width/level = 400/40) (a) Two 20-mm^2^ areas of brain parenchyma were encircled as regions of interest (ROIs), and the mean CT number (CT#_M_) and its standard deviation (SD_B_) (Hounsfield Unit, HU) were determined for the ROIs. The standard deviation (SD, HU) of the mean CT number (CT#_M_) within the ROI over the target region (ROI_M_) and background (ROI_B_) (b) The brain level.

Two 20-mm^2^ areas of brain parenchyma were encircled as regions of interest (ROIs), as shown in Fig 2, to determine the mean CT number (CT#_M_) and its SD_B_ (Hounsfield Unit, HU). The SD HU of the mean CT number (CT#_M_) within the ROI over the target region (ROI_M_) and background (ROI_B_) as in Fig 2 was calculated to evaluate the relationship between the contrast-to-noise ratio (CNRs) and noise in CT images [Eq (1)].


$$CNR=\frac{{CT\#}_{M}-{CT\#}_{B}}{{SD}_{B}}$$ (1)

### Dosimetry estimation

The CT dose volume index CTDI_vol_ describes the average radiation dose on the scanned area, measured in a standard test phantom. This test phantom is an acrylic cylinder with the diameter of 16 cm (head). The weighted dose length product (DLP_W_) is the product of CTDI_vol_ and the length (d) of the scanned area. The newest scanners usually provide dose displays for both CTDI_vol_ and DLP_W_. The accuracy of the dose display reading is verified by regular measurements, as part of the quality assurance of the scanners [Eq (2)].


DLP=CTDI·d (2)

ED was calculated for head CT by multiplying DLP, the individual dose report, with the dose conversion factor (k) in mGy^-1^cm^-1^ as Eq (3), as recommended by the International Commission on Radiological Protection (ICRP) Publication 96 [30].


ED=DLP(mGy∙cm)×k (3)

Where, for the brains of 1-, 5-, 10-year-old APs, the scan length weighting factors (k) for 80 kVp were 0.0067, respectively; those for 100 kVp were 0.004, respectively; and those for 120 kVp were 0.0032, respectively [30].

All inter-relationships among CTDI_vol_, DLPs, EDs and SDs, and CNRs in each age group can be explained by Eqs (1), (2), and (3).

### Statistical analysis

Statistical analysis was performed using GraphPad Prism 6 (Graphpad Software Inc., La Jolla, CA, USA). A one-way analysis of variance and post-hoc tests were performed to determine whether any significant difference (*p* < .05) existed in (1) SDs, (2) CNR, and (3) ED.

## Results

### CTDI_vol_, DLPs, EDs, SDs, and CNRs of the three AP brains

**Fig 3:**
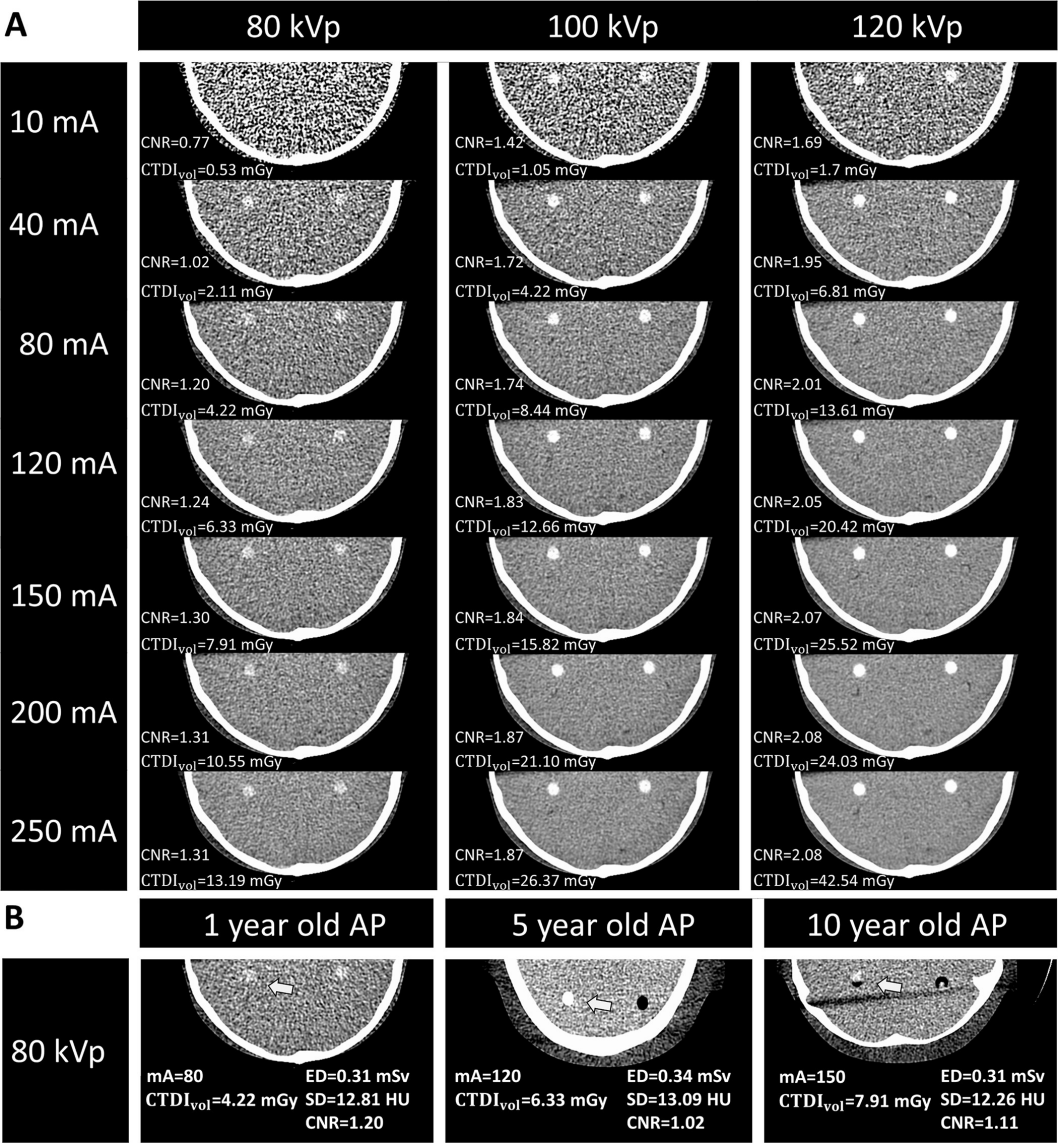
Axial images and the related SDs, CNRs, and CTDI_vol_ of the 1-year-old child phantom head exposed to three series (80, 100, and 120 kVp) of tube voltages/currents (A) and those of the 1-, 5-, and 10-year-old children phantom heads exposed to the optimal voltage/current, i.e. 80 kVp/80 mA, 80 kVp/120 mA, and 80 kVp/ 150 mA, respectively (B). Abbreviations: SD, standard deviation; CNRs, contrast noise ratios; CTDI_vol,_ volume computerized dose index.

The optimal values of CTDI_vol_ (mGy), DLP (mGy·cm), and conversion factors for 1-year-olds were: CTDI_vol_/DLP = 4.22/61.59, with k = 0.0067; for 5-year-olds: CTDI_vol_/DLP = 6.33/97.14, with k = 0.004; and for 10-year-olds: CTDI_vol_/DLP = 7.91/129.33, with k = 0.0032, respectively. Table 1 shows findings from scans of the three AP heads with 21 combinations of tube voltages/currents. Reduction of tube voltages from 120 to 80 kVp and currents from 250 to 10 mA in the three APs’ heads caused parallel reductions in CTDI_vol_, DLPs, EDs and CNRs, but increased SDs. Increase in age caused parallel increases in SDs and DLPs and reductions in EDs and CNRs; however, it had the same effects on CTDI_vol_ (Table 1). Note that the CTDI_vol_ for 1- (Fig 3A), 5-, and 10-year-old (Table 1) AP heads ranges from 0.53 to 42.54. The ranges of changes in DLPs, SDs, EDs and CNRs are further shown (Table 2). There were three possibly optimal SDs and CNRs with each tube voltage/current for the APs of each age (Table 3); the least kVp/mA for 1-, 5-, and 10-year-old APs were 80 kVp/80 mA, 80 kVp/120 mA, and 80 kVp/150 mA, respectively.

**Table 3:** Optimal kVp/mA associated with optimal SDs, EDs, and CNRs of CT images were obtained by scanning 1, 5, and 10-year-old anthropomorphic phantom (AP) heads (Please refer to Table 1).

	kVp	mA	EDs (mSv)	SDs (HU)	CNRs
1-year-old	80	80	0.31	12.81	1.20
	100	40	0.34	12.49	1.72
	120	40	0.61	10.76	1.95
5-year-old	80	120	0.34	13.09	1.02
	100	80	0.52	11.79	1.50
	120	40	0.48	12.77	1.69
10-year-old	80	150	0.31	12.26	1.11
	100	80	0.37	11.04	1.65
	120	40	0.34	12.30	1.90

SD = standard deviation; CNR = contrast-to-noise ratio; ED = effective dose.

### The least tube voltages/currents and CT images

The typical experiment (Fig 3A) for the 1-year-old AP revealed that tube voltage/current combinations of 80 kVp/80 mA, 100 kVp/40 mA, and 120 kVp/40 mA yielded CT images with SDs of 12.81, 12.49, or 10.76 HU, all of which were sufficient for diagnosis. Although higher tube voltages/currents still slightly improved the CT images with smaller SDs and greater EDs, the improvement in image quality was not markedly better. The same protocol was used for evaluations in 5- and 10-year-old APs and the 80 kVp/120 mA and 80 kVp/150 mA combinations, respectively, showed optimal results. Fig 3B reveals that optimal axial images of 1-year-old (left), 5-year-old (middle), and 10-year-old (right) APs were obtained with tube voltage/current combinations of 80 kVp/80 m, 80 kVp/120 mA, and 80 kVp/150 mA, respectively. The corresponding CTDI_vol_ and DLP values for these optimal combinations were as follows: for 80 kVp/80 mA, CTDI_vol_ = 4.22 mGy and DLP = 61.59 mGy·cm; for 80 kVp/120 mA, CTDI_vol_ = 6.33 mGy and DLP = 97.14 mGy·cm; and for 80 kVp/150 mA, CTDI_vol_ = 7.91 mGy and DLP = 129.33 mGy·cm.

### Relationships between CNRs and tube voltages/currents

**Fig 4:**
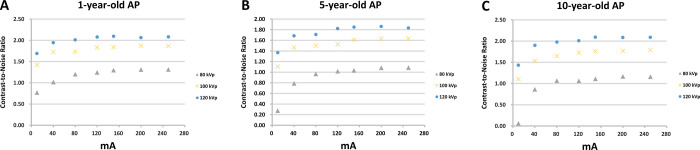
The relationships between contrast-to-noise ratio (CNR) and exposure voltages/currents for 1-, 5-, 10-year-old AP heads (A, B, and C, respectively). (right) Phantom induced by 80 kVp/80 mA, 80 kVp/120 mA, and 80 kVp/150 mA. The assessments of contrast-to-noise ratio (CNR) were performed through the entire anatomical region of interest where inserts were clearly visible (arrows). ED = effective dose; SD = standard deviation; CNR = contrast-to-noise ratio.

Fig 4 shows the relationships between CNRs and tube voltages/currents for 1-, 5-, 10-year-old AP heads (A, B, and C, respectively). Although the CNR increased with either voltage or current, the CNR curves tended to be suppressed by the higher voltage and flattened by higher currents. This indicated that the rate of increase in the CNR or image quality was limited by the higher voltage or current. Table 4 shows the advantage of using the least voltage/current.

**Table 4:** Comparisons of the CNR_LVC_ and CNR_VVC_ with CTDI_LVC_ and CTDI_VVC_ for the three AP groups.

Ages	1-year-old AP	5-year-old AP	10-year-old AP
Parameter			
LVC (kVp/mA)	80/80	80/120	80/150
VVC (kVp/mA)	120/250	120/250	120/250
CTDI_vol_ (mGy)			
CTDI_VVC_/CTDI_LVC_	42.54/4.22	42.54/6.33	42.54/7.91
Times	10.10	6.72	5.37
Contrast-to-noise ratio (CNR)			
CNR_VVC_/CNR_LVC_	2.08/1.20	1.86/1.02	2.09/1.11
Times	1.73	1.82	1.88

Abbreviations: LVC, least voltage/current; VVC, Vendor-recommended voltage/current; CNR_LVC_ and CNR_VVC,_ the LVC-induced CNR and the VVC-induced CNR; CTDI_LVC_ and CTDI_VVC,_ the LVC-induced CTDI and the VVC-induced CT

## Discussion

### Major findings

We scanned the heads of three AP and found that reductions of tube voltages/currents caused parallel reductions in CTDI_vol_, DLPs, EDs, and CNRs and increases in SDs, and vice versa (Table 1). Table 2 provides a more concise summary of these data to demonstrate that those tube voltages/currents induced ranges of changes in SDs, CNRs, and EDs. The simplification of these findings in Figs 3 and 4 and Table 3 yielded the most important contribution of the present investigation, namely the optimal images for 1-, 5-, and 10-year-old APs were respectively obtained with voltage/current combinations of 80-kVp/80-mA, 80-kVp/120-mA, and 80-kVp/150-mA. Fig 4 also shows the relationships between CNR and tube voltages/currents for 1-, 5-, 10-year-old AP heads (A, B, and C, respectively), demonstrating that the CNR increased with either voltage or current, and the rate of increase in CNR curves tended to be suppressed by higher voltages and flattened by higher currents. Table 4 shows the advantage of using these optimized voltage/current combinations. We are expecting that the clinical translation of our findings in the future will optimize CT imaging for diagnosis while reducing the radiation impact in children as much as possible.

### Novelty and importance of the findings

We demonstrated that increases in tube voltages/currents induced increases in EDs and CNRs, but reductions in SDs were associated with increased image quality (Table 1). These findings are generally consistent with other investigations [31, 32], which nevertheless did not identify the tube voltages/currents providing optimal CT images for diagnosis in pediatric clinics [33]. Figs 3 and 4 and Table 3 demonstrate that the optimal axial images of the 1-year-old (left), 5-year-old (middle), and 10-year-old (right) APs were respectively obtained with voltage/current combinations of 80-kVp/80-mA, 80-kVp/120-mA, and 80-kVp/150-mA. Our present findings thus confirmed our hypothesis that optimal tube voltages/currents providing diagnostic CT images while avoiding excessive radiation exposure could be identified. In particular, the selected voltages/currents were much smaller than those used commonly in clinics or recommended by the vendor [33, 34]. This issue was novel, important, and convenient, and thus deserved investigation.

### The least and optimal voltage/current

To avoid the impact of greater EDs on patients, “optimal” voltages/currents that produce optimal CNRs, EDs, and SDs while yielding optimal image quality sufficient for diagnoses, are important. Because voltages/currents greater than the optimal values identified herein did not yield markedly better images (Figs 3 and 4), we recommended acquisition of CT images only with voltage/current values of 80 kVp/80 mA, 80 kVp/120 mA, and 80 kVp/150 mA for 1-, 5-, 10-year-old APs, respectively (Figs 3 and 4). Because these tube voltages/currents induced SDs, respectively, of 12.81, 13.09, and 12.26 HU, with small EDs of 0.31, 0.34, and 0.31 mSv, and small yet reasonable CNRs of 1.20, 1.02, and 1.11, these voltage/current values and their induced values were expediently defined optimal.

Whether the much smaller voltages/currents (refer to “***Novelty and importance of the findings”***) can be useful in making the image quality sufficient for diagnosis under clinical conditions is not known. Thus, the optimal values defined herein may be slightly modified further to meet specific requirements; for example, radiologists can slightly increase the optimal CNR value to about 1.5 from 1.20, 1.02 and 1.11 by using higher voltages of 100 and 120 kVp, or currents of 80–160 mA (Figs 3 and 4).

### Advantages of using the optimal voltage/current

Table 4 shows the advantages of using the optimal voltage/current values. The vendor-recommended voltage/current were as high as 120 kVp/250 mA and induced CTDI_VVC_ of 42.54 mGy for all age groups, while the optimal values induced CTDI_LVC_ of 4.22, 6.33, and 7.91 mGy for 1-, 5- and 10-year APs (Table 4). On the other hand, the vendor-recommended values yielded CNR values of 2.08, 1.86, and 2.06, respectively for 1-, 5-, and 10-year-old APs, while the optimal voltage/current values yielded CNR values of 1.20, 1.02, and 1.11, respectively, for the three age groups (Table 4). These findings suggest that using the optimal values instead of the vendor-recommended values improved the image quality (CNR) by 1.73, 1.82 or 1.88 times while reducing the radiation dose (CTDI_vol_) by 10.1, 6.72, and 5.37 times, respectively, for the three age groups. Thus, the radiation dose was much more markedly reduced while image quality was relatively less attenuated when the optimal values were used.

### Current technology

Iterative reconstruction (IR) can automatically adjust to yield proper image for diagnosis with less radiation exposure [35], and automatic tube current modulation (ATCM) is available to reduce radiation exposure dose [23]. However, these modalities are not widely available, and hospitals not equipped with these modalities can determine the optimal tube voltage/current while referring to our present investigation. Furthermore, although our primary objective was to determine the optimal voltage/current, there is scope for further optimization. For example, studies have not determined whether the use of IR or ATCM with the optimal values can further enhance image quality and reduce radiation exposure in the pediatric clinic. We do not know whether using IR or ATCM referring to the LVC or OVC would further reduce the exposure dose.

### Need for advanced investigations

In addition to abovementioned investigations covering IR or ATCM, other issues also need to be investigated, including proper adjustments for children aged between 1–5 years or those aged between 5–10 years, proper adjustment based on head sizes among children, and further optimization for different brain parenchymal tissues or different brain diseases [36]. Considering the clinical implications, further large-scale clinical translational assessments are certainly needed. The clinical translation of our findings needs to be validated by additional research and data and will require a multidisciplinary effort.

### Usage of tube voltages/currents parameters

We would like to clarify the reason for using scanning tube voltages of 80, 100, or 120 kVp with tube currents of 10, 40, 80, 120, 150, 200, or 250 mA. Our scanner was only equipped with voltages of 80, 100, and 120 kVp; for adults, higher voltages are generally used, and for children, 80–120 kVp was commonly used and sufficient [37]. Since currents of >350 mA are used for adults, we arbitrarily estimated that currents less than 250 mA would be sufficient for children.

### Limitations

The APs were meant to simulate pediatric patients with standard body size and tissue composition, which limits the generalization of the results to a population with heterogeneous body or disease types. This study was therefore limited to conventional nonenhanced head CT for varying indications such as headache, brain edema, brain injury, cerebral hemorrhage, and brain tumor [38–40]. Second, the values identified in this study cannot be conclusively considered optimal for children till they have been validated in translational studies in pediatric clinics.

## Conclusions

The optimal voltage/current values that induced optimal brain image quality associated with SDs, CNRs, and EDs are novel and essentially important, because they may eventually become sufficient for CT diagnosis with less radiation exposure to the child’s head, once the findings are clinically translated. The advantage of using these values was that the radiation dose (CTDI_vol_) was much more markedly reduced while image quality (CNR) was relatively less attenuated when using these optimal values.
